# Dangers from Ligature of Main Arteries

**Published:** 1918-03-09

**Authors:** 


### PARALYSIS FOLLOWING ARTERIAL INJURIES.

### Dangers from Ligature of Main Arteries.

The wide variety of injuries which now come under review in military hospitals offers many opportunities for new and original observations. By the deadly art of war experiments are being made on the human body, and from these lessons may be learned in reference both to physiological and to pathological doctrine. A conspicuous illustration of this statement is to be found in the case of wounds inflicted on the hinder part of the brain and in the light which such injuries have thrown on the exact position of the visual centres. Disputes on this question have long existed, and they have not been concluded by vivisection experiments made on the higher animals. Indeed, upon such experiments different authorities have founded quite different conclusions. Now, from materials gathered during the present war, we are in possession of the results of hinder brain injuries inflicted upon man himself, and one result is to provide us with confident and precise information relative to the exact situation on the cerebral cortex of the nerve centres which are concerned in the act of vision. Such a conclusion is obviously of high physiological interest and is not without value to the practical physician. The outcome of these and kindred experiences must in the meanwhile await an assured summary. There is no doubt it will attain large proportions when the medical history of the war comes to be written.

An interesting contribution relating to some of the effects produced by injury of one or other of the main arteries, and from the pen of Captain Harold Burrows (*British Medical Journal*, January 16), deserves recognition from the point of view above defined. The effects to which Captain Burrows specially draws attention are of a paralytic order and may include both loss of muscular power and anæsthesia. Hitherto, while these consequences of arterial trauma have been observed, they have usually been attributed either to injury of nerve trunks running in the neighbourhood of the wounded artery or to "functional" disturbances. The first of these two explanations Captain Burrows puts definitely out of court, for he quotes experiences in which the paralytic phenomena were present in regions supplied by nerves which most certainly were not involved in the injury to the artery. Regarding the "functional" explanation there is less room for competent demonstration. In some of the cases described the sensory loss was not limited to the regions on the distal side of the wound, and this, it is admitted, does lend some colour to the suggestion of "functional" disturbance. Captain Burrows endeavours to meet such a position by remarking that to call the anæsthesia "functional" does not carry us any further, which in a sense is true enough. But this is to challenge a very wide doctrine, for it would exclude the "functional" interpretation of any of the phenomena of disease, and until some effective substitute is found this doctrine may be said to hold the field. A more impressive argument against the functional nature of the anæsthesia following arterial injury is Captain Burrows' observation that this anæsthesia is not found in other injuries such as fractures, wounded joints, and so on. Manifestly, "functional" disturbances would, at least *a priori*, be expected here as much as in lesions of the arteries. But as the variety of anæsthesia known as "stocking" anæsthesia is limited to the latter there is a fair presumption that a "functional" interpretation of it may well be doubted.

At first sight it might be concluded that motor and sensory paralysis, following damage to a main artery, were due to obstruction of the vessel and to consequent defective blood supply in the area of its distribution; and without question this is sometimes the case. Indeed, ischæmic paralysis following injury of a considerable artery or due to sustained pressure as by splints or bandages is well known, and some of Captain Burrows' cases would seem to belong to this group. Thus, in these the calibre of the artery was completely obliterated, as shown by the absence of pulse in the distal vessels; but in another group—and it is on these he lays special stress—the distal pulse was present, and therefore it was certain that the injured vessel remained more or less pervious. In both groups there was paralysis, but in the former the muscles, as is usual in ischæmic paralysis, were found hard and inelastic to the touch, while with an unobliterated artery the paralysis was of the flaccid variety. Another difference is that in the ischæmic group the anæsthesia, while of the "glove" or "stocking" pattern, was strictly confined to the portion of the limb distal to the wound, while when the distal pulse was present the loss of cutaneous sensibility, at least in some instances, extended higher up the limb and was found on the proximal side of the injury. There is, therefore, a strong case for the argument that the flaccid paralysis and sensory loss found in cases of arterial injury not associated with blocking of the vessel cannot be attributed to a cessation of the blood flow. Captain Burrows suggests that the fundamental cause in these cases is damage to the arterial wall or to its sheath, and that the flaccid paralysis and anæsthesia are reflex consequences of such damage. An explanation of this order was a frequent refuge for many difficulties some twenty or thirty years ago. But nowadays the fashion has changed, and perhaps it has changed too violently. In any event Captain Burrows argues his case on the lines already explained, and his claim is not without attraction. He urges also that his observations bear upon surgical practice in so far as they give a warning against too ready a ligature of a main artery for the arrest of hæmorrhage, and invite a search for the bleeding vessel in the hope that this may be a branch rather than the chief vessel. Even when this hope is disappointed, suture of the rent or the temporary use of a Tuffier tube is to be preferred to ligature and to the risks which this involves.

